# Potential Role of Circulating miR-103a, miR-145 and miR-191 as Diagnostic Biomarkers for Ulcerative Colitis and Crohn’s Disease

**DOI:** 10.3390/ijms262411927

**Published:** 2025-12-11

**Authors:** Aleksandra Górecka, Celina Kruszniewska-Rajs, Joanna Gola, Tomasz Romańczyk, Marcin Romańczyk, Katarzyna Komosinska-Vassev

**Affiliations:** 1 Department of Clinical Chemistry and Laboratory Diagnostics, Faculty of Pharmaceutical Sciences in Sosnowiec, Medical University of Silesia in Katowice, 41-200 Sosnowiec, Poland; 2 Department of Molecular Biology, Faculty of Pharmaceutical Sciences in Sosnowiec, Medical University of Silesia in Katowice, 41-200 Sosnowiec, Poland; 3 H-T. Medical Center—Endotherapy, Clinic of Gastroenterology, Silesian Academy, 40-555 Katowice, Poland

**Keywords:** inflammatory bowel disease, ulcerative colitis, Crohn’s disease, miR-103a, miR-145, miR-191, biomarker

## Abstract

Inflammatory bowel disease (IBD), including ulcerative colitis (UC) and Crohn’s disease (CD), is a group of chronic inflammatory disorders characterized by alternating episodes of flares and clinical remission, often leading to intestinal fibrosis. MicroRNAs (miRNAs) are small non-coding RNAs that regulate, among other processes, cell proliferation, inflammation, and fibrosis, all of which are crucial in IBD pathogenesis and healing. Given their role in these mechanisms, miR-103a, miR-145, and miR-191 were selected as promising candidates for IBD diagnostic biomarkers. Serum expressions of miR-103a, miR-145, and miR-191 were analyzed in 47 IBD patients and 30 healthy controls. Expressions were quantified using qPCR and normalized to miR-375-3p. All analyzed miRNAs were significantly upregulated in both UC and CD compared to healthy controls. ROC curve analysis revealed miR-103a as the most promising biomarker, with AUC = 0.893 in UC and AUC = 0.905 in CD. Moreover, miR-103a demonstrated excellent sensitivity and specificity, 89.3% and 80% in UC, and 84.2% and 83.3% in CD, respectively. miR-191 also effectively differentiated UC patients from healthy individuals (AUC = 0.848; sensitivity 89.3%; specificity 80%). Comparable results of diagnostic indicators were obtained in the CD group, however, with lower sensitivity (73.7%). miR-145 showed good ability in differentiating both UC and CD patients with high sensitivity (85.7%; 84.2%) and satisfactory specificity (66.7%; 63.3%). The obtained results indicate the promising diagnostic potential of circulating miR-103a, miR-145, and miR-191 for both UC and CD.

## 1. Introduction

Inflammatory bowel disease (IBD) is a group of chronic inflammatory disorders, including predominantly ulcerative colitis (UC) and Crohn’s disease (CD). The pathogenesis of both UC and CD is complex and involves abnormal immune response, disruption of intestinal barrier integrity, gut dysbiosis, and environmental and genetic factors. Despite some similarities in pathogenesis, UC and CD differ in terms of disease extent and location, therapeutic management, as well as possible complications. IBD is a lifelong condition characterized by recurrent episodes of disease exacerbation manifested by abdominal pain, diarrhea, rectal bleeding, nausea, weight loss, and fatigue. Moreover, chronic inflammation in IBD may lead to serious complications, including intestinal perforation with massive bleeding, toxic megacolon, fistula, abscesses, and strictures. The occurrence of mentioned complications is dependent not only on the type of IBD (UC vs. CD), but also on diagnosis time and accuracy, selection of therapeutic approach, and precise monitoring of disease activity [1,2,3,4,5]. Diagnosis is typically based on a combination of clinical symptoms, laboratory tests, and endoscopic examination. The most commonly used laboratory biomarkers are C-reactive protein (CRP) and fecal calprotectin (FC). However, their diagnostic utility remains suboptimal: CRP is a general biomarker of systemic inflammation, and does not accurately reflect the intestinal inflammation, whereas FC, although sensitive, lacks specificity, as it cannot reliably differentiate IBD from other gastrointestinal diseases [6,7]. Considering the limitations of existing diagnostic methods, there is a serious need to identify new biomarkers that could support rapid and precise IBD diagnoses. Moreover, such biomarkers could enable non-invasive, repeatable, and accurate monitoring of disease activity during implemented treatment. The use of the mentioned biomarkers could limit the development of disease activity and its possible complications, resulting in better life quality and limiting surgical interventions in IBD patients.

MicroRNAs (miRNAs) are valid regulators of various biological processes, such as cell differentiation, proliferation, apoptosis, and inflammation. miRNAs degrade complementary mRNAs or suppress their translation, resulting in reduced or ineffective protein synthesis. A single miRNA may target hundreds of mRNAs, and collectively miRNAs are estimated to regulate over 30% of all protein-coding genes. Therefore, it is not surprising that miRNAs are recently being investigated not only as diagnostic biomarkers, but also as therapeutic agents in numerous diseases such as cancer, diabetes, cardiovascular disease, and IBD [8,9,10]. Due to miRNAs taking such a crucial role in the regulation of various biological processes, the aim of this study is to evaluate the potential utility of selected miRNAs in the diagnosis and monitoring of IBD.

The miRNAs selected for this study were miR-103a, miR-145, and miR-191. These miRNAs were selected given their association with inflammatory response, intestinal barrier integrity, and fibrosis, all of which processes play a valid role in IBD development. In fact, in an animal model of septic liver injury, miR-103a significantly decreased the release of TNF-α, IL-1β, and IL-6, whose role is well documented in IBD. These proinflammatory cytokines are engaged in not only the development and maintenance of the inflammatory process, but also in the disruption of the intestinal barrier integrity [11,12]. The significance of miR-103a in IBD may be confirmed by the fact that increased expression of miR-103a was observed in intestinal biopsies collected from CD patients with regard to the controls. miR-103a was also engaged in the fibrotic process in not only CD, but also other diseases, including cystic fibrosis, non-alcoholic fatty liver disease, and hypertensive nephropathy [13]. Significant role in IBD progression may also be played by miR-145, whose decreased expression was noted in animal models of colitis and intestinal mucosa of CD patients [14]. Interestingly, myofibroblasts with higher expression were associated with more intense tissue repair, which may indicate the beneficial effect of miR-145 on IBD progression [15]. Moreover, miR-145 was found to support the intestinal barrier integrity, and its disruption is common in IBD. The last of the analyzed miRNAs is miR-191. This miRNA is associated with the inflammatory process as it was found to increase the expression of proinflammatory cytokines such as TNF-α and IL-6 and activate the NF-κB pathway, often associated with IBD pathogenesis [11,16,17]. miR-191 may also play a significant role in the remodeling of the extracellular matrix (ECM), since it regulates the expression of metalloproteinases (MMPs) and their inhibitors [16].

Considering the crucial role of miR-103a, miR-145, and miR-191 in key processes related to IBD development, assessment of their expression may provide great utility in clinical IBD practice. Therefore, the aim of this study is to evaluate the role of circulating miR-103a, miR-145, and miR-191 in IBD diagnosis, as well as further differential diagnosis to UC and CD. Another aim of this study is to assess the usefulness of the miRNAs serum profile in monitoring the activity of both UC and CD.

## 2. Results

### 2.1. Characteristics of Subjects Enrolled in the Study

This study included a total of 47 patients with inflammatory bowel disease (IBD), including 28 with ulcerative colitis (UC) and 19 with Crohn’s disease (CD), as well as 30 healthy controls. The clinical characteristics of these patients, together with healthy controls, are presented in Table 1. The UC group comprised 16 females and 12 males, with a mean age of 41.4 years and a mean disease duration of 7.9 years. The UC patients were predominantly (60.7%) provided with biological anti-inflammatory treatment, whereas 21.4% were treated with conventional anti-inflammatory drugs and 17.9% with small-molecule drugs. The disease activity was evaluated in UC patients using the partial Mayo scale (pMayo), for which median values equaled 0 points. The majority of the UC group (60.7%) were identified with clinical remission, while mild, moderate, and severe disease were observed in 17.9%, 14.3% and 7.1% of patients, respectively. The median CRP level in the UC group equaled 2.7 mg/L, with 75% of patients presenting normal CRP levels (defined as <5 mg/L). The CD group included 7 females and 12 males, with an average age of 34.9 years and a mean disease duration of 8.1 years, which were comparable to the UC group. Similarly to the UC group, the majority of CD patients (63.2%) were provided with biological anti-inflammatory treatment, while 15.8% received conventional anti-inflammatory drugs and 21.1% were given small-molecule drugs. The disease activity in CD patients was evaluated using Crohn’s Disease Activity Index (CDAI), with a median score of 57.0 points. Clinical remission was noted in 78.9% of patients, while the remaining 21.1% were classified as having moderate disease activity. The mean CRP concentration was 2.27 mg/L, and 84.2% of CD patients presented normal CRP values. The control subjects included in this study were selected in order to correspond with the age of UC and CD patients. Therefore, the mean age of the control group was 40.6 years. The control group included 19 females and 11 males. Moreover, according to the inclusion and exclusion criteria for this group, control individuals were not hospitalized in 3 years prior to study, did not receive any treatment, and presented normal results of routine laboratory tests (including lipid profile, fasting glucose, and CRP concentrations, liver enzyme activity, and complete blood count with differential).

### 2.2. Circulating Expression of miR-103a, miR-145,miR-191 in Patients with Ulcerative Colitis, Crohn’s Disease, and Healthy Individuals

**Figure 1:**
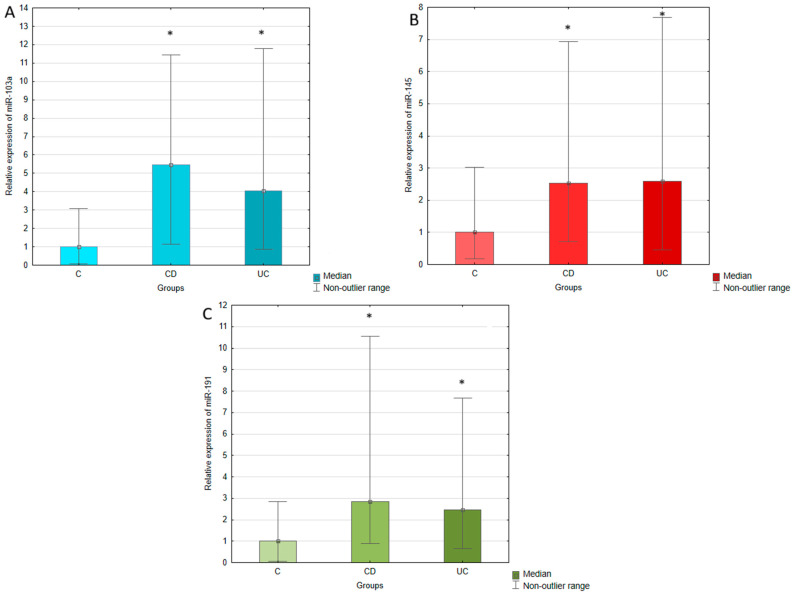
Relative expression of miR-103a (**A**), miR-145 (**B**), and miR-191 (**C**) in ulcerative colitis (UC), Crohn’s disease (CD), and control (C) groups. The results are presented as median fold change in relation to the control group. *, *p* < 0.05 with regard to the control group.

The expression of miR-103a, miR-145, and miR-191 was measured in the serum of IBD patients (both UC and CD) and healthy individuals using RT-qPCR. The expression of analyzed miRNAs was normalized to miR-375, which was selected as an endogenous control. The relative expression of miR-103a, miR-145, and miR-191 was calculated using the 2^−∆Ct^ method and compared between UC, CD, and control groups, which results are presented in Table 2 and Figure 1. The conducted statistical analyses revealed that serum expressions of all tested miRNAs were significantly upregulated in UC and CD patients compared to the healthy controls. The most pronounced difference was observed in the case of miR-103a, whose expression in UC patients was four times higher than in healthy individuals. In CD patients, this increase was even greater, achieving a 5.5-fold elevation compared to the control group. miR-145 expression was similar in UC and CD groups, presenting a 2.6-fold and 2.5-fold upregulation in relation to the controls. The expression of miR-191 was increased 2.5-fold in UC and 2.8-fold in CD with regard to healthy individuals. No significant difference was observed in serum expression of miR-103a, miR-145, and miR-191 between patients with UC and CD.

### 2.3. Circulating miR-103a, miR-145, miR-191 Expression, as Potential Biomarkers of Ulcerative Colitis and Crohn’s Disease

**Figure 2:**
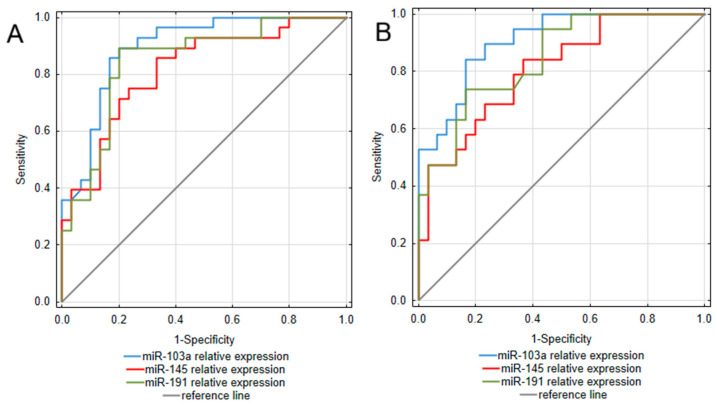
ROC curve analysis of miRNAs utility as diagnostic biomarkers of ulcerative colitis (**A**) and Crohn’s disease (**B**).

To assess the diagnostic value of the analyzed miRNAs, a receiver operating characteristic (ROC) curve analysis was performed. The obtained results are presented in Table 3 and Figure 2. Considering that the most pronounced difference between IBD patients and controls was observed in miR-103a expression, it is not surprising that this miRNA occurred as the most promising among the analyzed biomarkers for both UC and CD. Assessments of miR-103a expression enabled to differentiation of UC patients from healthy individuals with excellent ability, yielding an area under the curve (AUC) of 0.893 (95% CI: 0.811–0.975). This test was also characterized by high sensitivity (89.3%) and specificity (80.0%), together with great positive and negative predictive values (80.6% PPV; 88.9% NPV). In a group of CD patients, miR-103a measurements presented similarly high values of diagnostic characteristics, allowing for great differentiation of CD patients from healthy individuals with an AUC of 0.905 (95% CI: 0.825–0.985) and remarkable sensitivity (84.2%) and specificity (83.3%). miR-191 also occurred as a promising biomarker differentiating UC patients from healthy individuals, with an AUC yielding 0.848 (95% CI: 0.745–0.95). Moreover, miR-191 measurements presented equally great values of diagnostic characteristics as miR-103a in the UC group. In the CD group, miR-191 measurements were characterized by great specificity and NPV; however, compared to UC, this test presented lower sensitivity and thus, a greater risk of false-negative diagnosis in the CD group. miR-145 measurements also proved useful in differentiating both UC and CD patients from healthy individuals, with AUC yielding 0.815 (95% CI: 0.706–0.925) in UC and 0.805 (95% CI: 0.683–0.928) in CD. Considering greater values of sensitivity and NPV compared to specificity and PPV, in both UC and CD groups, this test could be more prone to generate false positive diagnoses.

### 2.4. Correlation of miR-103a, miR-145, and miR-191 Expression with Clinical Characteristics

Correlation analysis between circulating miR-103a, miR-145, and miR-191 levels and clinical parameters (disease activity, CRP, treatment time, and disease duration) was analyzed in UC and CD patients. No significant correlations were observed between the expression of tested miRNAs and disease activity, assessed by partial Mayo score in UC and CDAI scale in CD. The expression of miRNAs did not differ between patients with clinical remission and those with active disease in either the CD or UC groups. miRNA profiles did not vary between UC patients with mild, moderate, or severe disease activity. These results suggest that, although the evaluated miRNAs demonstrated strong diagnostic potential, they are not suitable markers for monitoring short-term fluctuations in disease activity. Moreover, CRP concentrations were assessed in both UC and CD patients in order to evaluate the intensity of inflammation; however, no significant relationships were found between circulating miRNAs and CRP profiles. Additionally, no treatment-related differences in serum miRNA expression were observed among patients receiving conventional anti-inflammatory, biological, or small-molecule therapies. The expression of tested miRNAs was not associated with disease or treatment duration, which may suggest that miR-103a, miR-145, and miR-191 upregulation is stable in long-term UC and CD. Moreover, the expression of tested miRNAs did not differ between biological, conventional anti-inflammatory, and small-molecule drugs. Significant correlations were, however, noted between the expression of tested miRNAs. The most pronounced associations were found between miR-103a and miR-191 serum expression in both UC (r = 0.922, *p* < 0.0001) and CD patients (r = 0.915, *p* < 0.0001), with very strong correlations being found. Other strong and significant correlations were observed between the expression of miR-103a and miR-145 in UC (r = 0.752, *p* < 0.0001) and CD (r = 0.758, *p* < 0.0005) groups. The associations between the expression of miR-145 and miR-191 were also evaluated as strong and significant in both UC (r = 0.707, *p* < 0.0001) and CD (r = 0.703, *p* < 0.001). These correlations between tested miRNAs might reflect a shared mechanism of up-regulation, overlapping targets, or an up-regulatory feedback loop linking them.

## 3. Discussion

The present retrospective and cross-sectional study identified miR-103a, miR-145, and miR-191 as significantly upregulated circulating miRNAs in IBD patients compared to healthy controls. The most pronounced difference was observed in the circulating profile of miR-103a, whose expression was 5.5 and 4 times higher in CD and UC patients compared to healthy individuals. miR-103a profile has not yet been studied in the serum of IBD patients; however, our results may be confirmed by the study of Qian et al. [13]. In that study, miR-103a expression was elevated in intestinal biopsies collected from CD patients compared to normal intestinal tissue. miR-103a upregulation was associated with intestinal fibrosis, as its enhanced expression was noted in fibroblasts and correlated with the degree of fibrosis in CD patients. In fact, Qian et al. indicated that miR-103a is able to activate fibroblasts by targeting tumor growth factor β receptor 3 (TGFBR3) and enhancing Smad 2/3 phosphorylation, promoting fibrotic remodeling [13,14]. This profibrotic action of miR-103a through TGFBR3 blockade was also confirmed in different diseases, including thyroid-eye disease (TED), as it was demonstrated by Xie et al. [15]. In that study, exposition of TED orbital fibroblasts to TGF-β increased already upregulated miR103a expression. miR-103a overexpression led to increased cell vitality, as well as elevated vimentin and fibronectin levels, while miR-103a inhibition decreased the observed effect. These results may indicate a vital role of miR-103a in the vicious cycle of TGF-β-mediated fibrosis during IBD. In that cycle, miR103a targets TGFBR3, resulting in upregulation of TGF-β signaling, while TGF-β may increase the expression of miR-103a in fibroblasts. Given that the average IBD duration in our study was approximately 8 years and patients were predominantly in remission, it is possible that miR-103a overexpression was related to ongoing intestinal fibrosis. Unfortunately, we are unable to compare miR-103a serum expression with the degree of intestinal fibrosis or microscopic healing, as endoscopic examinations were not performed in the included IBD patients. Most individuals were in clinical remission, and there was no medical indication to conduct an invasive procedure. We recognize that histological evaluation would provide meaningful insights; therefore, the lack of these assessments constitutes an important limitation of our study. Nevertheless, miR-103a is not only related to fibrosis, as its possible immunosuppressive role is also being indicated. In a mouse model of septic liver injury, Chen et al. found that miR-103a treatment inhibited the release of proinflammatory cytokines, including IL-6, IL-1β, and TNF-α [11]. Moreover, in Li et al.’s study, miR-103a overexpression was noted to decrease the level of IL-6, TNF-α, and CRP in the pneumonia cell model [16]. IL-6, IL-1β, and TNF-α are key proinflammatory cytokines contributing to the intestinal inflammation in IBD; therefore, miR-103a may also play a potential immunosuppressive role in IBD [11,12]. miR-103a overexpression may suppress the release of proinflammatory cytokines, resulting in the amelioration of intestinal inflammation and CRP concentrations. Thus, an increase in miR-103a in both UC and CD groups in our study may have contributed to the observed remission and correct CRP levels. Moreover, some studies indicate miRNAs regulate not only inflammatory pathways, but also the composition of the intestinal microbiome. Interestingly, these interactions are suggested to be bidirectional, which may be of great interest considering that gut dysbiosis is one of the factors contributing to IBD development [18]. For example, Rossi et al. [19] reported that eradication of *H. pylori* was associated with increased expression of the miR-103 family, while Assmann et al. [20] demonstrated a negative correlation between *Bacteroides eggerthii* abundance and circulating miR-103a-3p levels. Therefore, the elevated miR-103a levels observed in our patients may also reflect microbiome alterations typical of IBD. Future studies including simultaneous microbiome analysis and cytokine profiling would be necessary to verify this hypothesis. Considering miR-103a association with fibrosis and inflammatory processes, together with its marked overexpression in UC and CD groups compared to healthy individuals, this biomarker may be remarkably useful in IBD diagnosis and monitoring. Therefore, ROC curve analysis was performed to evaluate the diagnostic potential of miR-103a in both UC and CD. miR-103a expression profile was the most effective among all tested miRNAs in the differentiation of IBD patients from healthy individuals. Serum miR-103a profile presented high values of sensitivity and specificity in both UC and CD patients, indicating low occurrence of both false positive and negative diagnoses. Moreover, as diagnostic indicators were similar in both groups of patients, miR-103a is similarly effective in UC and CD diagnoses. Therefore, serum miR-103a expression may support the diagnostic process for both UC and CD with equal accuracy.

In this study, elevated miR-145 expression was observed in IBD patients compared to healthy individuals, with a 2.6-fold and 2.5-fold increase in UC and CD groups, respectively. In contrast to our results in Pekow et al.’s [21] study, miR-145 expression was decreased in intestinal biopsies collected from UC patients with regard to healthy individuals. Intestinal downregulation of miR-145 during IBD was further confirmed by Li et al. [22]. In that study, miR-145 expression was decreased in inflamed intestinal tissue compared to non-inflamed and normal intestinal tissue in both UC and CD patients. These results are in contrast with our findings; however, they may suggest that higher miR-145 expression could be related to mucosal healing and clinical remission. Therefore, as patients included in our study were predominantly in remission, observed miR-145 upregulation may be related to the achievement of mucosal healing, being the key aim of IBD therapy. Unfortunately, as there are no studies evaluating both serum and intestinal miR-145 profiles in IBD patients during active disease and remission, it is not clear if observed in our study serum miR-145 upregulation is common in active and inactive disease or indicates mucosal healing. Interestingly, in Li et al.’s [22] study, they analyzed epithelial, stromal, and immune cells collected from IBD patients, where myofibroblasts were the ones with the highest miR-145 expression in both UC and CD patients, indicating their valid role in IBD development and healing. Moreover, myofibroblasts expressing miR-145 were characterized with a mesenchymal phenotype, enhanced proliferation, activation of Wnt/β-catenin signaling, and were a regulator of epithelial cell proliferation and tissue regeneration [23]. Moreover, the beneficial effect of miR-145 overexpression in IBD could be confirmed by the study of Zhuang et al. [24]. In that study, treatment with miR-145 in a mouse model of TNBS-induced colitis was found to improve intestinal inflammation, histological score of colon tissue, and body weight. Moreover, miR-145 enhanced the integrity of the intestinal barrier by upregulating claudin 8 expression. Considering the crucial role of miR-145 in mucosal healing and intestinal barrier integrity, its serum profile may prove useful in IBD diagnosis. In fact, conducted ROC curve analysis conducted revealed a very good ability of miR-145 in differentiating UC patients from healthy individuals with great sensitivity, however, lower specificity. miR-145 also effectively differentiated patients with CD from healthy individuals with similar sensitivity as in the UC group; however, lower specificity and PPV. Given the values of specificity and PPV, miR-145 appears to be more prone to overdiagnoses, especially in the CD group. Therefore, its assessments may be more beneficial for UC rather than CD diagnoses.

The last of the analyzed miRNAs-miR-191—also upregulated in both UC and CD patients compared to healthy individuals. Our results may be confirmed by the Paraskevi et al. [25] study, in which increased serum miR-191 expression was noted in patients with CD. In contrast to our study, Paraskevi et al. noted no significant difference in miR-191 profile between UC patients and healthy individuals. Unfortunately, Paraskevi et al. did not evaluate the disease activity; therefore, it is not clear whether the observed difference was caused by divergent UC activity between the studies. In fact, Gu et al. [26], using human endothelial cells, indicated that proinflammatory cytokines such as TNF-α and IL-6 may increase the miR-191 expression in a dose-dependent manner. Thus, the observed differences in miR-191 expression between our and Paraskevi’s findings may be a result of higher TNF-α and/or IL-6 levels among UC patients in this study. As neither Paraskevi et al. nor we evaluated the TNF-α and IL-6 levels in UC patients, this hypothesis cannot be confirmed. Another explanation of observed differences may be the epigenetic mechanisms regulating miRNA expression. Indeed, He et al. [27] indicated that hypomethylation at the miR-191 locus resulted in miR-191 overexpression in human hepatocellular carcinoma. Therefore, it is possible that the methylation pattern of the miR-191 locus in UC patients is variable or dependent on disease activity. Moreover, in that study, miR-191 overexpression downregulated epithelial markers such as E-cadherin or pan-cytokeratin, while upregulating mesenchymal markers including vimentin and N-cadherin. These results suggest a significant role of miR-191 in promoting epithelial-to-mesenchymal transition, a process closely related to fibrosis. Thus, the observed miR-191 upregulation in IBD patients in this study may contribute to intestinal fibrosis. Furthermore, Gu et al. [26] reported that miR-191 overexpression decreases MMP-1 and MMP-9, while increasing TIMP-1 mRNA levels. During IBD, MMP-9 is increased in both the intestinal tissue and serum, correlating with disease activity. Elevated MMP-9 concentration is related to ECM degradation, tissue injury, and disruption of intestinal barrier integrity. The MMP-9 inhibitor, TIMP-1, plays a protective role during acute inflammation; however, it becomes profibrotic in chronic IBD. Consequently, increased TIMP-1 levels are associated with the fibrosis process. In contrast, MMP-1, as collagenase, plays an anti-fibrotic role [27,28,29]. Therefore, during IBD, miR-191 overexpression may suppress MMP-1 and MMP-9, while enhancing TIMP-1 expression, leading to disrupted MMP/TIMP balance. This imbalance may result in attenuation of intestinal inflammation; however, it also promotes intestinal fibrosis. Considering the valid role of miR-191 in IBD, its diagnostic potential was also evaluated in this study. Conducted ROC curve analysis revealed that miR-191 presented a great ability to differentiate patients with UC from healthy individuals. This differentiation was characterized by equally high sensitivity and specificity as miR-103a assessments in UC patients. Thus, measurements of miR-191 in the UC group occurred as the second most promising biomarker analyzed in this study. Serum miR-191 profile also effectively differentiated CD patients from healthy individuals, however, with lower sensitivity. These results indicate a greater risk of false-negative diagnoses in the CD group compared to UC and highlight a more effective role of miR-191 in UC diagnoses. Considering the significant role of miRNAs in regulating numerous proteins, and the increasing number of studies evaluating their diagnostic utility, miRNA analyses may eventually be introduced into routine clinical practice. In this study, to ensure the reproducibility of the presented results, miRNA extractions and quantification were performed using widely available test kits without modification of the protocols. However, we recognize that before implementation in clinical practice, each stage of the miRNA analysis must be validated, and medical personnel must be properly trained. Therefore, despite their innovative and promising potential, miRNA analysis is not yet ready to be immediately introduced into clinical practice, which is one of the limitations of this study.

Additionally, apart from their diagnostic potential, assessments of serum miR-103a, miR145, and miR-191 expressions could be beneficial in screening for IBD-related colorectal cancer. Long-term IBD patients, especially UC, are at greater risk of developing colorectal cancer (CRC) [30]. Therefore, it is extremely important to monitor not only the activity of the IBD, but also the occurrence of CRC. Considering that the miR-103a, miR-145, and miR-191 expressions are altered during CRC, these miRNAs may not be used only in IBD diagnosis, but also in CRC screening. Patients with CRC usually present decreased miR-145, with increased miR-103a and miR-191 expressions, thus miR-145 downregulation with simultaneous miR-103a and miR-191 upregulation may identify IBD-related CRC patients among all IBD patients [31,32,33,34,35,36]. This hypothesis should be further confirmed in future studies, as among our patients, none were diagnosed with IBD-related CRC. Considering the limited number of patients recruited in our research, future studies should include larger, multicenter cohorts encompassing a broader demographic and clinical diversity of IBD patients. Such studies would enhance statistical power and allow evaluation of miRNA fluctuations across different disease stages, treatment durations, and time intervals. This observation may, however, present another advantage of miR-103a, miR-145, and miR-191 as IBD biomarkers.

Interestingly, serum expression of the analyzed miRNAs significantly correlated with each other. Mentioned correlations were evaluated as very strong between miR-103a and miR-191 and strong between miR-103a and miR-145, as well as between miR-145 and miR-191. It is noteworthy that the strength of these correlations was comparable between UC and CD groups, indicating shared regulatory mechanisms underlying the observed relationship among the analyzed miRNAs. Possible explanations of these correlations may be overlapping transcriptional activation, shared functional response, epigenetic alterations, and a common cellular source. Given that all miRNAs are related to the fibrotic process, a shared functional response may be one of the possible explanations. In fact, miR-103a and miR-191 are both referred to as onco-miRNA, regarding their role in regulating cell cycle and viability. Zhang et al. [36] indicated that miR-191 not only induced cell transition from G1 to S phase, but also increased cell viability and decreased apoptotic rate. Similar observations were made by Fasihi et al. [32] regarding miR-103a, which overexpression promotes transition to the S phase of the cell cycle and reduces apoptotic rate. The observed strong correlation between miR-103a and miR-191 might arise from their mutual function. A similar observation can be made in the case of miR-103a and miR-145. In Li [22] and Fasihi et al.’s [32] studies, overexpression of miR-145 and miR-103a was related to enhanced activity of Wnt/β-catenin signaling, indicating their related role in cell proliferation and tissue regeneration. miR-195 and miR-145 also share similar functions, as miR-145 and miR-191 overexpression was related to enhanced epithelial-to-mesenchymal transition [22,27]. Considering the strong relationship between analyzed miRNAs and their shared regulative role in tissue regeneration, fibrosis, and tumorigenesis, miR-103a, miR-145, and miR-191 may fill a valid role not only in IBD diagnosis but also in evaluating new therapeutic strategies in IBD. Elucidating the functional impact of miR-103a, miR-145, and miR-191 in IBD in future studies would provide a deeper understanding of their clinical relevance. Integrating miRNA expression data with transcriptomic and proteomic profiling in future studies may help identify downstream molecular pathways influenced by these miRNAs in IBD.

## 4. Materials and Methods

### 4.1. Study Population

This study employed a retrospective, observational, and cross-sectional design. A total of 77 subjects were enrolled in this study, including 28 patients with ulcerative colitis (UC), 19 patients with Crohn’s disease (CD), and 30 healthy individuals. All patients with UC and CD were diagnosed, treated, and monitored at the H-T. Medical Center in Tychy, Poland. The diagnoses of ulcerative colitis or Crohn’s disease were made based on endoscopic examination, laboratory tests, and clinical symptoms. Disease activity was evaluated using the partial Mayo index in ulcerative colitis patients and the Crohn’s disease activity index (CDAI) in Crohn’s disease patients. The inclusion criteria for participation in the study group were age over 18 years and a confirmed diagnosis of ulcerative colitis or Crohn’s disease. Subjects for the control group were recruited during routine appointments conducted as part of mandatory periodic occupational health examinations. These visits were unrelated to any symptoms or medical concerns. The inclusion criteria for the control group were age over 18 years and normal results in routine laboratory tests, including lipid profile, fasting glucose, and CRP concentrations, liver enzyme activity, and complete blood count with differential. The exclusion criteria for the control group were hospitalization up to 3 years before the study and ongoing treatment. Moreover, the control subjects were selected to correspond to the age and sex distribution of patients with ulcerative colitis and Crohn’s disease. The biological material used in this study was venous blood, which was collected once from patients with IBD and healthy individuals.

### 4.2. Methods

miRNA was extracted from 200 μL of serum of IBD patients and healthy individuals using miRNeasy Serum/Plasma Advanced Kit (Qiagen, Valencia, CA, USA). Then, cDNA was synthesized from the isolated RNA using miRCURY LNA RT Kit (Qiagen, Valencia, CA, USA). The cDNA was used in a qPCR reaction to determine the expression of selected miRNAs, being: hsa-miR-103a-3p, hsa-miR-145-5p, and hsa-miR-191-5p, as tested miRNAs, while hsa-miR-375-3p and U6 snRNA were used as candidate reference miRNAs. The qPCR reaction was conducted using miRCURY LNA miRNA PCR Assay and miScript Primer Assays (Qiagen, Valencia, CA, USA) on Light Cycler 480 System (Roche Life Science, Basel, Switzerland). RNA isolation, reverse transcription, and qPCR reactions were performed according to manufacturers’ protocols. To monitor the efficiency and correctness of RNA isolation and cDNA synthesis, UniSp4 miRCURY LNA miRNA PCR Assay and UniSp6 miRCURY LNA miRNA PCR Assay (Qiagen, Valencia, CA, USA) were used as the controls. All reactions were performed in duplicate. RNA isolation, reverse transcription, and qPCR reactions were performed according to manufacturers’ protocols.

The relative expression of analyzed miRNAs (miR-103a, miR-145, and miR-191) was defined using the 2^−∆Ct^ method, after normalization with reference miRNA. The expression of miR-375-3p and U6 snRNA was tested for the reference miRNA. The expression of miR-375-3p was the most stable across the tested subjects and was selected as the reference miRNA.

### 4.3. Statistical Analyses

All statistical analyses in this study were performed using STATISTICA software, version 13.3 (StatSoft, Cracow, Poland). The Shapiro–Wilk test was applied to assess the normality of data distribution. The differences in circulating miRNA profiles between analyzed groups were evaluated using Student’s *t*-test for normally distributed data or Mann–Whitney U test for non-normally distributed data. Receiver operating characteristic (ROC) curve analysis was conducted to determine the diagnostic potential of selected miRNAs. Correlation between serum profiles of analyzed miRNA with disease activity and CRP values was assessed using Pearson’s or Spearman’s correlation coefficients, depending on the data distribution. In all conducted statistical analyses, a *p* value of <0.05 was considered statistically significant.

## 5. Conclusions

The findings of this study indicate that serum miR-103a, miR-145, and miR-191 are significantly elevated in both ulcerative colitis and Crohn’s disease. Among them, miR-103a and miR-191 exhibited the strongest diagnostic utility in the effective differentiation of UC patients from healthy individuals. Moreover, miR-103a occurred as the most promising biomarker of CD, also presenting great sensitivity and specificity. Measurements of miR-145 expression presented great utility in differentiating UC and CD patients, but with lower specificity, indicating a greater risk of false-positive diagnoses. Therefore, the assessments of circulating miR-103a, miR-145, and miR-191 expressions may support the diagnostic process in both UC and CD, improving the diagnostic accuracy.

## Figures and Tables

**Table 1:** Clinic characteristics of the study and control groups.

Parameter	UC	CD	C
N	28	19	30
Sex (females/males)	16/12	7/12	19/11
Age [years]	41.4 ± 13.9	34.9 ± 11.3	40.6 ± 11.4
Disease duration [years]	7.9 ± 6.0	8.1 ± 4.8	-
Treatment			
Conventional anti-inflammatory treatment	6	3	-
Biological treatment	17	12	
Small-molecule drugs	5	4	
Disease activity:			
Indices values	pMayo: 0 (0–3)	CDAI: 57.0 (39.0–125.5)	-
Clinical remission	17	15	
Mild	5	0	
Moderate	4	4	
Severe	2	0	
CRP [mg/L]	2.70 (1–5.40)	2.27 (0.94–3.57)	-
RBC [×10^6^/μL]	4.4 (4.02–5.10)	4.7 ± 0.50	-
HGB [g/dL]	13.57 ± 1.91	14.35 ± 1.55	-
MCV [fL]	90.2 (85.8–95.4)	91.85 ± 6.73	-
PLT [×10^3^/μL]	294.5 ± 82.4	261.1 ±86.8	-
WBC [×10^3^/μL]	7.01 ± 2.33	5.82 ± 1.80	-
Neutrophils [×10^3^/μL]	4.03 ± 1.75	3.71 ± 1.17	-
Lymphocytes [×10^3^/μL]	2.10 ± 0.84	1.39 ± 0.56	-
Monocytes [×10^3^/μL]	0.57 ± 0.18	0.55 ± 0.22	-
Eozynophils [×10^3^/μL]	0.15 (0.11–0.22)	0.07 (0.05–0.11)	-
Basophils [×10^3^/μL]	0.04 (0.03–0.05)	0.02 (0.02–0.03)	-
ALT [U/L]	25.34 ± 10.23	21.04 ± 14.86	-
AST [U/L]	23.68 ± 7.32	22.73 ± 8.64	-

Data is presented as mean ± standard deviation in case of normally distributed data and median with interquartile range in non-normally distributed data. ALT, alanine aminotransferase; AST, aspartate aminotransferase; C, control group; CD, group of patients with Crohn’s disease; CRP, C-reactive protein; HGB, hemoglobin; MCV, mean corpuscular volume; N, number of individuals; PLT, platelets; pMayo, partial Mayo scale index; RBC, red blood cells; UC, group of patients with ulcerative colitis; WBC, white blood cells.

**Table 2:** Fold change and *p* value of the analyzed miRNA between the study and control groups.

miRNA	UC		CD	
	Fold Change	*p* Value	Fold Change	*p* Value
miR-103a	4.0	0.00000	5.5	0.00000
miR-145	2.6	0.00004	2.5	0.00037
miR-191	2.5	0.00001	2.8	0.00006

Fold change and *p* value were calculated using the relative miRNA expression (2^−∆Ct^) in each study group compared to the control group.

**Table 3:** ROC curve analysis of the analyzed miRNAs.

Analyzed miRNA	Analyzed Groups	AUC (95% CI)	Youden Index	Cut-Off [Relative Expression]	Sensitivity (%)	Specificity (%)	PPV (%)	NPV (%)
miR-103a	UC	0.893 (0.811–0.975)	0.69	25.02	89.3	80.0	80.6	88.9
	CD	0.905 (0.825–0.985)	0.68	26.54	84.2	83.3	76.2	89.3
miR-145	UC	0.815 (0.706–0.925)	0.52	5.88	85.7	66.7	70.6	83.3
	CD	0.805 (0.683–0.928)	0.48	5.06	84.2	63.3	59.3	90.5
miR-191	UC	0.848 (0.745–0.95)	0.69	25.11	89.3	80.0	80.6	88.9
	CD	0.843 (0.735–0.951)	0.57	36.5	73.7	83.3	73.7	83.3

AUC, area under the curve; CD, patients with Crohn’s disease; CI, confidence interval; NPV, negative predictive value; PPV, positive predictive value; UC, patients with ulcerative colitis.

## Data Availability

The original contributions presented in this study are included in the article. Further inquiries can be directed to the corresponding author.
